# Mitogenome analysis of a green tide forming *Ulva* from California, USA confirms its identity as *Ulva expansa* (Ulvaceae, Chlorophyta)

**DOI:** 10.1080/23802359.2018.1535859

**Published:** 2018-10-29

**Authors:** Jeffery R. Hughey, Kathy Ann Miller, Paul W. Gabrielson

**Affiliations:** a Division of Mathematics, Science, and Engineering, Hartnell College, Salinas, CA, USA;; b University Herbarium, University of California, Berkeley, CA, USA;; c Biology Department and Herbarium, University of North Carolina at Chapel Hill, Chapel Hill, NC, USA

**Keywords:** Bloom, holotype, rbcL, tufA, UPA

## Abstract

An unknown species of marine sea lettuce was observed forming green tides consecutive years from 2014 to 2016 in Seaside, California. This *Ulva* sp. was similar in thallus size and shape to *U. expansa*. To confirm this identification, whole genome sequencing was performed on the bloom-forming species of *Ulva* and the holotype specimen of *U. expansa*. The complete green tide *Ulva* mitogenome is 64,143 bp in length, contains 65 genes, and displays high gene synteny with *U. pertusa* Kjellman. The mitogenome was incomplete for the holotype of *U. expansa*, but the analysis yielded the mitoexome, plastid, and nuclear genetic markers. These data verify that the native *U. expansa* is responsible for the blooms in central California.

Green tides are blooming events of green algae that result from eutrophication and warming seawater (Fletcher 1996, Shi & Wang 2009, Yoshida et al. 2015, Gao et al. 2017). They have been reported worldwide and their occurrences, particularly members of the genus *Ulva*, are on the rise (Smetacek & Zingone 2013). During the month of July in consecutive years 2014–2016, green tides of *Ulva* at Seaside, California were observed. To determine the identity of this *Ulva* species, genome sequencing was performed on a 2014 collection and on the holotype of *U. expansa* (Setchell) Setchell & N.L.Gardner (type locality: Monterey, California). Plastid markers were also sequenced from the 2015 and 2016 green tide collections to confirm their identities.

DNA was isolated from 5 × 5 mm^2^ snippets from the holotype specimen of *U. expansa* (specimen voucher UC 98481) and from a herbarium specimen of the bloom-forming *Ulva* from 2014 (UC 2050480) following the protocols of Lindstrom et al. (2011) and Hughey and Gabrielson (2012). The holotype was processed in 2016 using 76 bp paired-end Illumina library construction and sequencing, and the modern material in 2018 using 150 bp Illumina sequencing by myGenomics, LLC (Alpharetta, GA). The data were assembled using the default *de novo* settings in CLC Genomics Workbench 11 (^®^2018 CLC bio, a QIAGEN Company, Waltham, MA), and by mapping reads and contigs from the *de novo* analysis against the mitogenomes of *U. linza*, *U. pertusa*, and *U. prolifera* using default settings in Geneious R8 (Biomatters Limited, Auckland, New Zealand). The genes were annotated with Blastx and NCBI ORFfinder. The mitogenome data were aligned to other *Ulva* species with MAFFT (Katoh & Standley 2013). The RaxML analysis was executed using complete mitogenome sequences at Trex-online (Boc et al. 2012) with the GTR + gamma model and 1000 fast bootstraps, then visualized with TreeDyn 198.3 at Phylogeny.fr (Dereeper et al. 2008).

The complete mitogenome of the bloom-forming *Ulva* is 64,143 bp in length and contains 65 genes. The mitogenome of the holotype of *U. expansa* was incomplete; however, the genomic analysis yielded its mitoexome (GenBank numbers MH730977-MH731006). The two *Ulva* genomes contain *cob*, 2 rRNAs, 3 *cox*, 3 *rpl*, 5 ATP synthases, 7 orfs, 8 *nad*, 9 *rps*, and 27 tRNAs. Gene content, organization, and length of the blooming *Ulva* was similar to *U. pertusa* (Liu et al. 2017). Phylogenetic analysis of the two mitogenomes indicates a sister relationship to *U. pertusa* (Figure 1). Analysis of *rbc*L sequences of the 2014–2016 bloom-forming *Ulva* from Seaside (GenBank numbers 2014 – MH730975, 2015 – MH730976, 2016 – MH746437) found three identical sequences, all differing from the holotype of *U. expansa* by only 2 bp. Comparison of plastid and nuclear markers of the holotype of *U. expansa* (GenBank numbers *tufa* – MH731007, UPA – MH731008, *rbc*L – MH731009; and SSU/ITS/LSU – MH730160) to the bloom-forming *Ulva* (GenBank numbers *tufa* – MH730973, UPA – MH730974, *rbc*L – MH730975; and SSU/ITS/LSU – MH730161) supports the conclusion that the bloom-forming *Ulva* from Seaside is the native central Californian species *U. expansa*.

**Figure 1.**
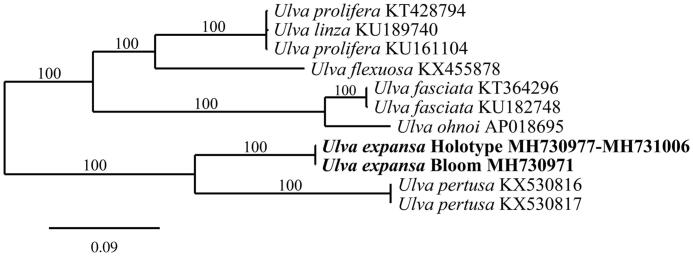
Maximum likelihood phylogram of the holotype of *Ulva expansa,* the green tide forming *U. expansa*, and related Ulvales mitogenomes. Numbers along branches are RaxML bootstrap supports based on 1000 nreps. The legend below represents the scale for nucleotide substitutions.
